# Therapeutic Effect of Chung-Pae, an Experimental Herbal Formula, on Acute Lung Inflammation Is Associated with Suppression of NF-**κ**B and Activation of Nrf2

**DOI:** 10.1155/2013/659459

**Published:** 2013-08-26

**Authors:** Kyun Ha Kim, Do-Hyun Kim, Nara Jeong, Kwan-Il Kim, Yong Ho Kim, Mei Lee, Jun-Yong Choi, Hee Jae Jung, Sung-Ki Jung, Myungsoo Joo

**Affiliations:** ^1^Division of Applied Medicine, School of Korean Medicine, Pusan National University, Yangsan 626-870, Republic of Korea; ^2^Division of Allergy, Immune and Respiratory System, Department of Internal Medicine, College of Oriental Medicine, Kyung Hee University, Seoul 130-701, Republic of Korea; ^3^College of Korean Medicine, Daegu Haany University, Daegu 706-060, Republic of Korea; ^4^Department of Korean Medical Science, School of Korean Medicine, Pusan National University, Yangsan 626-870, Republic of Korea; ^5^Department of Internal Medicine, Korean Medicine Hospital, Pusan National University, Yangsan 626-870, Republic of Korea

## Abstract

Acute lung injury (ALI) is an inflammatory disease with high mortality, but therapeutics against it is unavailable. Recently, we elaborated a formula, named Chung-pae (CP), that comprises four ethnic herbs commonly prescribed against various respiratory diseases in Asian traditional medicine. CP is being administered in aerosol to relieve various respiratory symptoms of patients in our clinic. Here, we sought to examine whether CP has a therapeutic effect on ALI and to uncover the mechanism behind it. Reporter assays show that CP suppressed the transcriptional activity of proinflammatory NF-**κ**B and activated that of anti-inflammatory Nrf2. Similarly, CP suppressed the expression of NF-**κ**B dependent, proinflammatory cytokines and induced that of Nrf2 dependent genes in RAW 264.7. An aerosol intratracheal administration of CP effectively reduced neutrophilic infiltration and the expression of pro-inflammatory cytokines, hallmarks of ALI, in the lungs of mice that received a prior intraperitoneal injection of lipopolysaccharide. The intratracheal CP administration concomitantly enhanced the expression of Nrf2 dependent genes in the lung. Therefore, our results evidenced a therapeutic effect of CP on ALI, in which differential regulation of the two key inflammatory factors, NF-**κ**B and Nrf2, was involved. We propose that CP can be a new therapeutic formula against ALI.

## 1. Introduction

Acute lung injury (ALI) is a severe inflammatory disease with substantial morbidity and mortality in human. Hallmarks of ALI include neutrophilic infiltration to the lung parenchyma, abnormal lung compliance, and impaired gas exchange [1–3]. While systemic inflammation by bacterial infection is the major cause of ALI, severe multiple trauma, aspiration pneumonia, and complications of mechanical ventilation often lead to ALI [1]. Despite extensive studies and clinical trials, therapeutic measures against the disease have been elusive [4].

Lipopolysaccharide (LPS), a cell wall component of Gram-negative bacteria, is known as a major activator of inflammatory response that leads to ALI. It binds to TLR4 to activate NF-*κ*B, which largely accounts for the production of proinflammatory cytokines including tumor necrosis factor-*α* (TNF-*α*) and interleukin (IL)-1, -6, -8, -10, -12, and -15 families [5]. These cytokines contribute to recruiting neutrophils to the lungs, where neutrophils clear up infectious agents. Since NF-*κ*B is also found to be chronically active in many other inflammatory diseases such as inflammatory bowel disease, arthritis, and gastritis [5], therapeutics strategies have been focused on attenuating NF-*κ*B activity and the production of Proinflammatory cytokines, which turns out to be ineffective [6]. On the other hand, accumulating evidence suggests that Nrf2 plays an essential role in protecting against various inflammatory diseases, including acute pulmonary injury, smoke-induced emphysema, and asthma [7–9]. Although Nrf2 is emerging as a potential therapeutic target for inflammatory diseases [9, 10], no effective therapeutics against ALI is available yet.

Several herbal medicinal formulas have been prescribed for the treatment of various lung diseases in Asian traditional medicine [11]. Therefore, it is possible that these formulas are a resource for the development of a new therapeutic measure against deadly respiratory diseases. Popular herbal formulas against respiratory diseases include Ma-Huang Tang that has been used against cough with rigor, Junghangshichae-tang against cough with phlegm and nausea, Gwackhyangjungki-san against cold with nausea and abdominal pain, and Kyeji-tang against cold with headache and fever [11]. Although each herbal formula is typically composed of several herbs, it contains a key herb that accounts for the efficacy of a formula. For instance, *Ephedrae Herba* is the key ingredient of Ma-Huang Tang; *Caryophylli Flos* is the key herb in Junghangshichae-tang; and *Pogostemonis (Agastachis) Herba *and *Zingiberis Rhizoma Crudus* are key herbs in Gwackhyangjungki-san and Kyeji-tang, respectively. In the hope of obtaining a herbal medicine effective on a broad spectrum of respiratory diseases, we formulated a new herbal medicine, named Chung-pae (CP), that is, composed of these key herbs contained in the four formulas. CP is being used as a complementary measure in the respiratory clinic at Kyung-Hee Oriental Medicine Hospital, Seoul, Korea, to relieve the symptoms of patients with dyspnea and cough. Although it seemed that CP helps improve the symptoms, experimental evidence supporting its effect is scarce. Here, we sought to examine whether CP has a therapeutic effect on respiratory diseases and, if it does, how CP exerts its effect. To this end, we used an LPS-induced ALI mouse model. Since CP is being administered in aerosol to the patients, we delivered CP in aerosol to the lung and determined the effect of CP on ALI in mice. To obtain an insight on the underlying mechanism, we tested whether CP affects the activities of Proinflammatory factor NF-*κ*B and/or anti-inflammatory factor Nrf2. Our findings show that aerosol delivery of CP to the lung effectively reduced the hallmarks of ALI, which was appeared to be associated with suppression of Proinflammatory factor, NF-*κ*B, and activation of anti-inflammatory factor, Nrf2.

## 2. Material and Methods

### 2.1. Preparation of the Water Extract of Chung-Pae

The herbs composing of Chung-pae (CP), shown in Table 1, were purchased from Kwang-Myoung-Dang herb store (Pusan, Republic of Korea) and identified by Professor J. Y. Choi (School of Korean Medicine, Pusan National University, Yangsan, Republic of Korea). The voucher specimen (number: pnukh004) is kept in the herbarium stock room of the School of Korean Medicine, Pusan National University. A crude decoction of CP was obtained by boiling 60 g of Chung-pae in 400 mL distilled water for 2 hours followed by filtration through 0.45 *μ*m filter. The resultant decoction was concentrated to 50 mL by a low-pressure evaporator and underwent freeze-drying processes to yield 6 g of powder. Appropriate amount of the powder was dissolved in phosphate buffered saline (PBS) prior to experiment.

### 2.2. Reagents and Antibodies

3-(4,5-dimethylthiazol-2-yl)-2,5-diphenyltetrazolium bromide, Sulforaphane, and *E. coli* LPS (serotype 055 : B5) for animal study were from Sigma Chemical Co. (St. Louis, MO, USA). TLR4-specific *E. coli* LPS was purchased from Alexis Biochemical (San Diego, CA, USA). 

### 2.3. Animals

Male C57BL/6 mice, inbred in a specific pathogen-free (SPF) facility, were purchased from Samtaco Bio Korea, Ltd. (Osan, Korea). Animals were housed in certified, standard laboratory cages and fed with food and water *ad libitum* prior to experiment.

### 2.4. ALI Mouse Model and Intrapulmonary Delivery of Chung-Pae

All experimental procedures followed the NIH of Korea Guidelines for the Care and Use of Laboratory Animals, and all the experiments were approved by the Institutional Animal Care and Use Committee of Pusan National University (protocol number: PNU-2010-00028). Mice, anesthetized by Zoletil (Virbac, Carros cedex, France), received a single dose of 10 mg/kg LPS or sterile saline via intraperitoneal (i.p.) route. At 2 h after i.p. LPS, either PBS or CP (5 and 20 mg/kg body weight) in 25 *μ*L of PBS was loaded in MicroSprayer Aerosolizer-Model IA-1C (Penn-Century, Wyndmoor, PA, USA) and delivered in aerosol to the lung via intratracheal (i.t.) under visual guidance. At 24 h after LPS treatment, mice were euthanized by CO_2_ gas. The trachea was exposed through midline incision and cannulated with a sterile 24-gauge intravascular catheter. Bilateral bronchoalveolar lavage (BAL) was performed by two consecutive instillations of 1.0 mL of PBS. Total cell numbers in BAL fluid were counted with hemocytometer and then centrifuged by a cytospin and stained for the differentiation of macrophages, lymphocytes, or neutrophils by Hemacolor (Merck, Darmstadt, Germany). Three hundred cells in total were counted, and one hundred of the cells in each microscopic field were scored. The mean number of cells per field was reported. For the analysis of lung tissue, mice were perfused with saline and the whole lung was inflated with fixatives. After paraffin embedding, 5 *μ*m sections were cut and placed on charged slides, and stained with hematoxylin and eosin (H&E) staining method. Three separate H&E-stained sections were evaluated in 200x microscopic magnifications per mouse.

### 2.5. Cell Culture

RAW 264.7 cells (American Type Culture Collection, Rockville, MD, USA) were cultured in Dulbecco's Modified Eagle's Medium (DMEM) containing L-glutamine (200 mg/L) (Invitrogen; Carlsbad, CA, USA) supplemented with 10% (v/v) heat-inactivated fetal bovine serum (FBS) and 100 U/mL penicillin and 100 *μ*g/mL streptomycin (Invitrogen; Carlsbad, CA, USA), and maintained in a humidified incubator at 37°C and 5% CO_2_ prior to experiment.

### 2.6. Microculture Tetrazolium (MTT) Assay

MTT assay was performed to evaluate the cytotoxicity of CP. RAW 264.7 cells (1.0 × 10^4^ cells/well) were treated with CP for 16 h, where MTT solution was added. After 4 h incubation in cell culture incubator, formazan crystals formed in viable cells were dissolved with DMSO, and the optical density (OD) of formazan was measured at 540 nm with a microplate reader. Cell viability was calculated as a percentage against the untreated. All experiments were performed three times independently.

### 2.7. Reporter Constructs, Reporter Cell Line, and Luciferase Assay

To estimate Nrf2 and NF-*κ*B transcriptional activity, we used reporter cell lines stably harboring an NQO-1/luciferase reporter and NF-*κ*B/luciferase reporter constructs [6, 7]. Luciferase activity was measured by a luciferase assay kit (Promega, Madison, WI, USA) per the manufacture's instruction and normalized by the amount of total proteins of the cell extract.

### 2.8. Isolation of Total RNA from Cells and RT-PCR

Total RNA was isolated from right lung homogenates with TRIZOL reagent (GeneAll, Korea) according to the manufacturer's instructions. Two micrograms of total RNA were reverse-transcribed by M-MLV reverse transcriptase (Promega). Target mRNA was quantified by using end-point dilution PCR, including three serial 1 to 5 dilutions (1 : 1, 1 : 5, 1 : 25, and 1 : 125) of RT products for PCR amplification. The level of GAPDH (glyceraldehyde-3-phosphate dehydrogenase) cDNA from each sample was used to normalize the samples for differences in PCR efficiency. For PCR amplification, TaqPCRx DNA polymerase (Invitrogen) and the manufacturer's protocol were used. Resultant cDNA was amplified by PCR with a set of specific primers. The forward and the reverse primers for NQO-1 were 5′-GCAGTGCTTTCCATCACCAC-3′ and 5′-TGGAGTGTGCCCAATGCTAT-3′; the primers for HO-1 were 5′-TGAAGGAGGCCACCAAGGAGG-3′ and 5′-AGAGGTCACCCAGGTAGCGGG-3′; the primers for GCLC were 5′-CACTGCCAGAACACAGACCC-3′ and 5′-ATGGTCTGGCTGAGAAGCCT-3′; the primers for IL-1*β* were 5′-GTGTCTTTCCCGTGGACCTT-3′ and 5′-TCGTTGCTTGGTTCTCCTTG-3′; the primers for TNF-*α* were 5′-CTACTCCTCAGAGCCCCCAG-3′ and 5′-AGGCAACCTGACCACTCTCC-3′; and the primers for GAPDH were 5′-GGAGCCAAAAGGGTCATCAT-3′ and 5′-GTGATGGCATGGACTGTGGT-3′. The reaction conditions were as follows: an initial denaturation at 95°C for 5 min followed by 28 cycles of denaturation for 30 sec at 95°C, annealing for 30 sec at 58°C and extension for 40 sec at 72°C with a final extension for 7 min at 72°C. Amplicons were separated in 1.5% agarose gels. GAPDH was used as internal controls to evaluate relative expressions of TNF-*α*, IL-1*β*, GCLC, HO-1, and NQO1. Relative expression of each gene over GAPDH was determined by densitometric analysis software ImageJ (Wayne Rasband, Research Services Branch, National Institute of Mental Health, Bethesda, MD, USA). Reactions were separated in 1.2% agarose gels in 1 × TBE buffer at 100 V for 30 min, stained with SYBR safe DNA gel stain (Invitrogen) and visualized under LED light.

### 2.9. Statistical Analysis

To compare the results among groups, one-way analysis of variance (ANOVA) tests with Tukey's post hoc test was used (with the assistance of InStat, Graphpad Software, Inc., San Diego, CA) (*P* values < 0.05 are considered significant). All experiments were performed at least three times independently.

## 3. Results

### 3.1. The Water Extract of Chung-Pae Suppresses the Transcriptional Activity of NF-*κ*B and the Expression of Proinflammatory Cytokines in RAW 264.7 Cells

**Figure 1:**
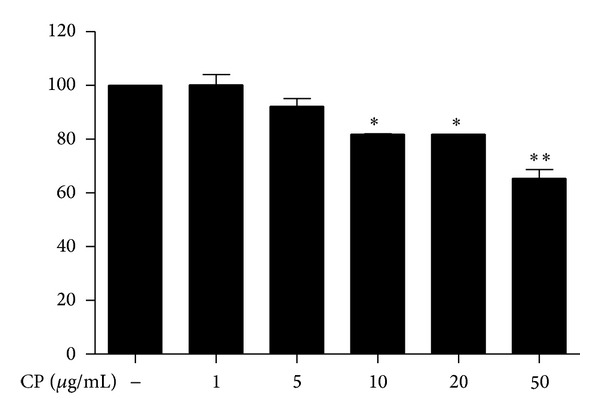
Cytotoxicity of Chung-pae. MTT assay was performed to measure the cytotoxicity of CP by using RAW 264.7 cells. Cells were treated with indicated amounts of CP for 16 hours prior to MTT assay. Data represent the mean ± SEM of three independent measurements. *P and **P were less than 0.05 and 0.01, respectively, compared to untreated, control group.

For the study, we prepared and used the water extract of CP. First, we tested whether CP has any cellular toxicity. RAW 264.7 cells, a murine macrophage-like cell line, were treated with various amounts of CP, from 1 *μ*g/mL to 50 *μ*g/mL. At 16 h after treatments, MTT assay was performed. As shown in Figure 1, CP showed a slight cytotoxicity within the range of 20 *μ*g/mL but a significant cytotoxicity at 50 *μ*g/mL. However, MTT assay at 12 h after treatments showed no cytotoxicity within the range of 20 *μ*g/mL, while 50 *μ*g/mL of CP did a significant cytotoxicity (data not shown). Therefore, we chose to use the amounts ranged from 5 *μ*g/mL to 20 *μ*g/mL of CP in this study.

**Figure 2:**
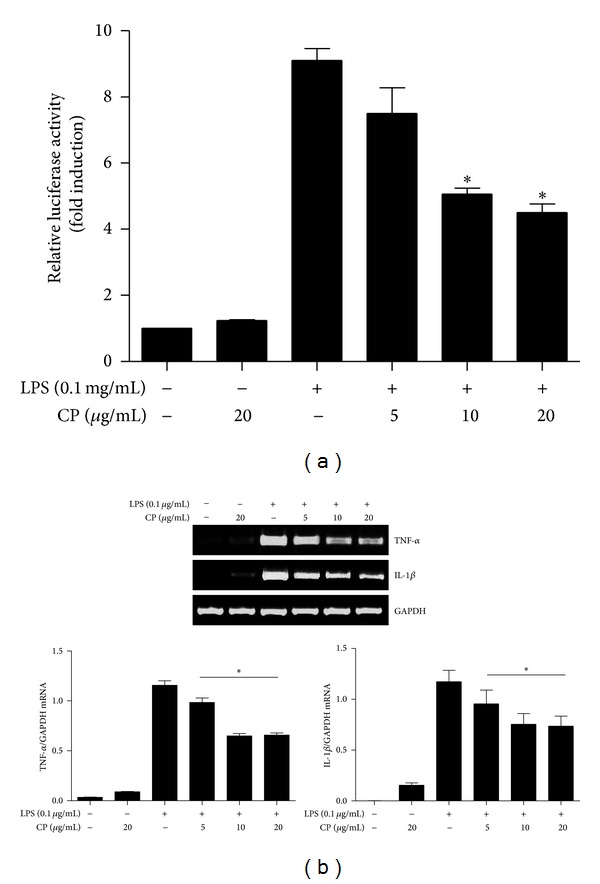
Chung-pae suppresses NF-*κ*B transcriptional activity and NF-*κ*B dependent gene expression. (a) The transcriptional activity NF-*κ*B was measured in an NF-*κ*B reporter cell line derived from RAW 264.7 cells. The cell line was pretreated with indicated amounts of CP for 16 h and then subsequently with TLR4 specific LPS (0.1 *μ*g/mL) for 8 h. Luciferase activity was normalized by the amount of total proteins in cell lysate. Treatment with 5 *μ*g/mL of CP was not statistically significant. *P was less than 0.05, compared to the LPS-treated. Data represent the mean ± SEM of three independent experiments. (b) RAW 264.7 cells were treated with CP and LPS as in (a). Total RNA was extracted and analyzed by semiquantitative RT-PCR for TNF-*α* and IL-1*β*. The intensity of each PCR band was measured by densitometric analysis (ImageJ), and the relative expression of each gene was calculated over GAPDH. *P was less than 0.05, compared to the LPS treated. Data are presented as the mean ± SEM of 3 separate experiments.

Next, we tested the possibility that CP exerts its effect by regulating NF-*κ*B activity, given that NF-*κ*B regulates expressions of Proinflammatory cytokines and chemokines including TNF-*α*, IL-1, 6, 8, 10, 12, 15, and MIP-1*α* [5]. To determine whether CP affects the transcriptional activity of NF-*κ*B, we took a RAW 264.7 cell line that stably harbors an NF-*κ*B-luciferase reporter construct [12] and treated it with different amounts of CP for 16 h, and subsequently treated with LPS (0.1 *μ*g/mL). At 8 h after LPS treatment, total cell lysate was prepared for luciferase assay. As shown in Figure 2(a), 10 *μ*g/mL or 20 *μ*g/mL of CP significantly reduced luciferase activity driven by activated NF-*κ*B, suggesting that CP suppresses the transcriptional activity of NF-*κ*B. To determine whether decrease of NF-*κ*B activity results in reduced expression of NF-*κ*B dependent genes, we performed similar experiments with RAW 264.7 cells and analyzed the expression of Proinflammatory cytokines governed by NF-*κ*B. As shown in Figure 2(b), expressions of IL-1*β* and TNF-*α* were similarly decreased by CP treatment. These results indicate that CP suppresses NF-*κ*B activity, contributing to suppression of inflammatory gene expression.

### 3.2. CP Activates the Transcriptional Activity of Nrf2 and Induces Expression of Nrf2-Dependent Genes in RAW 264.7 Cells

**Figure 3:**
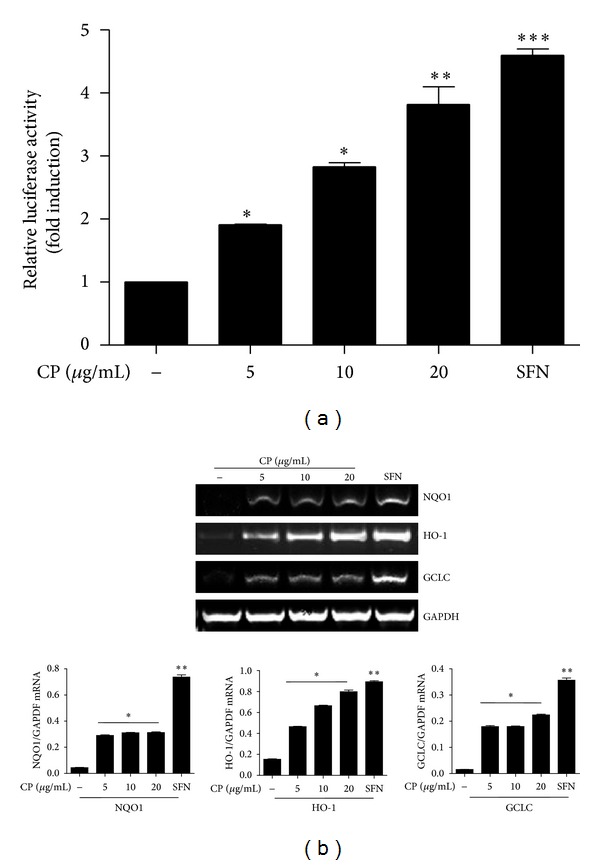
Chung-pae activates Nrf2 transcriptional activity and induces Nrf2 dependent genes. (a) An Nrf2 reporter cell line, derived from RAW 264.7 cells, was treated with SFN (5 mM) or the indicated amounts of CP for 16 hours. Luciferase activity was normalized by the amount of total proteins. Data are shown in the mean ± SEM of three independent measurements. *P, **P, and ***P were less than 0.05, 0.01, and 0.001, respectively, compared to untreated control. (b) RAW 264.7 cells were treated with CP, similar to (a). Total RNA was analyzed by semiquantitative RT-PCR for NQO-1, HO-1, and GCLC. The intensity of each PCR band was measured by densitometric analysis (ImageJ), and relative expression of each gene was calculated over GAPDH. *P and **P were less than 0.05, compared to the untreated. Data are presented as the mean ± SEM of 3 separate experiments.

Accumulating evidence suggest that Nrf2 is a master anti-inflammatory factor that prevents from acute lung inflammation [8, 13, 14]. Therefore, we tested the possibility that CP affects Nrf2 activity, contributing to the effect of CP. We used an Nrf2-luciferase reporter cell line derived from RAW 264.7 cells [15] and treated it with increasing amounts of CP (1, 5, 10, 20 *μ*g/mL). At 16 h after treatment, total cell lysate was prepared for luciferase assay. As shown in Figure 3(a), similar to treatment with sulforaphane (5 *μ*M), a well-documented Nrf2 activator [16], CP treatment increased the luciferase activity in a dose dependent manner, suggesting that CP activates the transcriptional activity of Nrf2. To determine whether activation of Nrf2 results in the expression of Nrf2 dependent genes, we performed similar experiments to determine whether CP treatment induces the expression of Nrf2 dependent genes. RAW 264.7 cells were treated with CP as described above, and total RNA of the treated cells was extracted for semiquantitative RT-PCR analyses of NQO-1, HO-1, and GCLC (Figure 3(b)), prototypical Nrf2 target genes [17, 18]. As shown in Figure 3(b), CP treatment induced the expression of Nrf2 dependent genes. Combined with CP suppressing NF-*κ*B, these results suggest that CP exert an anti-inflammatory function by both suppressing NF-*κ*B and activating Nrf2.

### 3.3. Intratracheal Delivery of CP Ameliorates Neutrophilic Infiltration to the Lung in an LPS-Induced ALI Mouse Model

**Figure 4:**
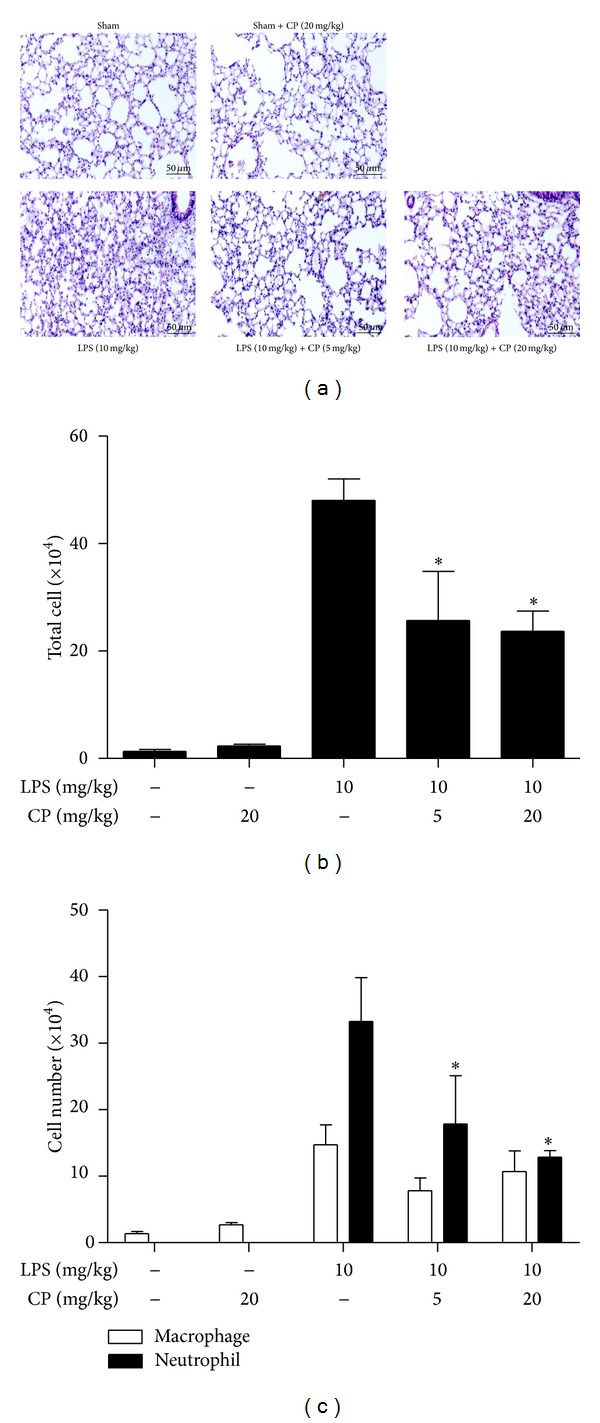
Aerosol intratracheal Chung-pae administration suppresses acute neutrophilic lung inflammation in LPS-induced ALI animal model. (a) H&E stained lung sections of C57BL/6 mice. C57BL/6 mice received sham (top panels) or an i.p. injection of LPS (bottom panels). At 2 h after the treatments, mice received 20 mg/kg (top 2nd panel and bottom 3rd panel) or 5 mg/kg (bottom 2nd panel) of CP in aerosol via trachea. At 24 h after LPS administration, the lungs of mice were analyzed by histological examination. Data are representatives of at least five different areas of a lung (200x magnifications). Total cells (b) and neutrophils and macrophages (c) in BAL fluid were scored. *P was less than 0.05, compared to the mice treated with LPS only. Data are presented as the mean ± SEM of 5 mice per group.

Since our results show that CP activated Nrf2, a key transcription factor that has been known to protect from acute lung injury (ALI) [14], we tested whether CP has a therapeutic effect on ALI. To this end, we set up an LPS-induced ALI mouse model. Mice received an i.p. LPS (10 mg/kg body weight) for the induction of lung inflammation. At 2 h after LPS injection, various amounts of CP were administered to the lungs of LPS-treated mice. Given that daily dose of CP to patients is 5 mg/kg body weight and that our results indicated that CP was effective in activating Nrf2 from 5 *μ*g/mL to 20 *μ*g/mL (Figure 3), we tested two different doses: 5 mg/kg, equivalent to the dose for patients, and 20 mg/kg of CP. Since CP is being prescribed in a form of inhalant, CP was loaded in a microsprayer and delivered in aerosol to the lung via trachea. At 24 h after LPS treatment, mice were euthanized, and the lungs were harvested for the analysis of the effect of CP on neutrophilic infiltration to the lung. As shown in Figure 4(a), H&E staining of lung sections shows that while controls received sham or CP (20 mg/kg) (*n* = 5/group) only maintained alveolar structure intact (top two panels), LPS-injected mice developed a characteristic lung structure due to inflammation (1st panel in the bottom). However, i.t. CP administration, either 5 mg/kg or 20 mg/kg, ameliorated the inflammatory lung structure (bottom 2 panels from left).

To determine whether CP regulates neutrophilic infiltration, we performed bronchoalveolar lavage (BAL) and counted the infiltrates in BAL fluid. As shown in Figures 4(b) and 4(c), while LPS administration increased the cellular infiltration to the lung, in which neutrophils were predominant (3rd columns from the left), both doses of CP significantly reduced the number of neutrophils in the lung (4th and 5th columns). Together, these results show that CP posttreatment relieved neutrophilic lung inflammation induced by LPS, suggesting that CP has a therapeutic effect on ALI.

### 3.4. Intratracheal Delivery of CP Reduces the Expression of Proinflammatory Cytokines and Activates That of Nrf2-Dependent Genes in the Lung

**Figure 5:**
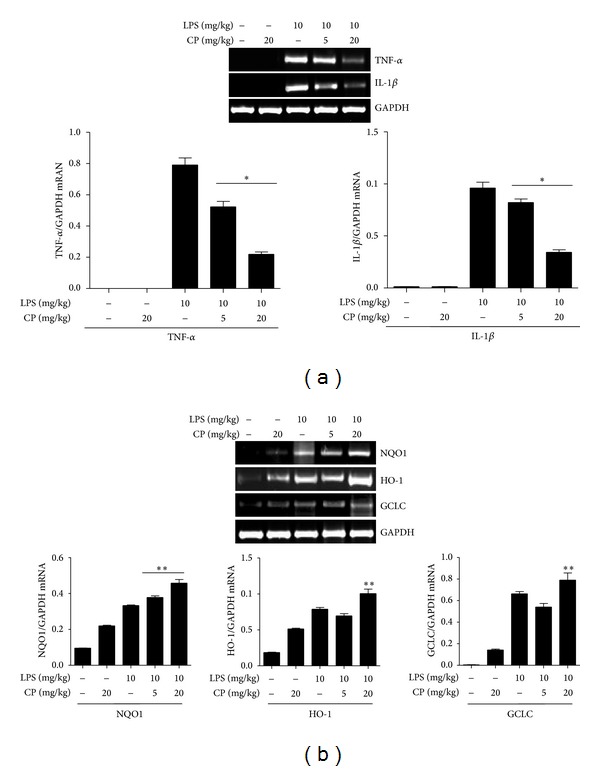
Aerosol intratracheal Chung-pae administration suppresses the expression of Proinflammatory cytokines and enhances the expression of Nrf2-dependent genes in the lungs of mice. Mice (*n* = 5/group) received 5 mg/kg or 20 mg/kg of CP 2 h after an i.p. LPS injection. At 24 h after LPS injection, the lungs of variously treated mice were harvested for semiquantitative RT-PCR analysis of Proinflammatory genes (a). The intensity of each PCR band was measured by densitometric analysis (ImageJ), and relative expression of each gene was calculated over GAPDH. *P was less than 0.05, compared to the mice treated with LPS only. (b) Similarly, the expression of Nrf2 dependent genes in the lung was analyzed. Mice, treated with 20 mg/kg of CP, increased the expression of Nrf2 dependent genes. Expressions of these genes were enhanced by CP. *P was less than 0.05, compared to untreated control, and **P was less than 0.05, compared to the LPS treated.

Since our results with macrophage implicated CP in suppressing NF-*κ*B activity and activating Nrf2, we tested whether the therapeutic effect of CP on lung inflammation in ALI mice is associated with suppressed NF-*κ*B and activated Nrf2. We treated mice as described in Figure 4 and harvested the lungs of mice (*n* = 5/group). Total RNA in the lung was extracted, quantitated, and analyzed by semiquantitative RT-PCR. As shown in Figure 5(a), expressions of Proinflammatory cytokine genes, such as TNF-*α* and IL-1*β*, were decreased by i.t. CP administration. Meanwhile, expressions of Nrf2 dependent genes, such as NQO-1, HO-1, and GCLC, were enhanced by i.t. CP administration (Figure 5(b)). Both suppression of the expression of Proinflammatory cytokines and enhancement of Nrf2-dependent gene expression became apparent when mice received a higher dose of CP. In any event, these results suggest that the therapeutic effect of CP is associated with suppression of NF-*κ*B and activation of Nrf2.

## 4. Discussion

In this study, we sought to obtain experimental evidence that CP has a therapeutic effect on ALI and to unveil underlying mechanisms for the effect. Since CP comprises four herbs that have been reported to have anti-inflammatory activities *in vitro,* we hypothesized that the effect of CP on respiratory symptoms is related to the anti-inflammatory activities exerted by its constituents. Our results show that CP suppressed neutrophilic infiltration to the lung and the production of Proinflammatory cytokines, hallmarks of ALI, in an LPS-induced ALI mouse model, which was associated with suppression of Proinflammatory transcription factor NF-*κ*B and activation of anti-inflammatory factor Nrf2. 

CP is a composite formula of *Ephedrae Herba, Caryophylli Flos, Pogostemonis (Agastachis) Herba, and Zingiberis Rhizoma Crudus*. In Asian traditional medicine, these herbs have been mainly prescribed for respiratory diseases, except *Caryophylli Flos.* For instance, *Ephedrae Herba* has been known to be effective in reducing wheezing, asthma, and edema;* Pogostemonis (Agastachis) Herba *is mainly used for cold and nausea in summer; and *Zingiberis Rhizoma Crudus* is mainly for common cold, nausea, and cough with phlegm [19]. On the other hand,* Caryophylli Flos *has been traditionally used for abdominal disorders including vomiting, hiccup, pain, diarrhea, and lack of appetite [19]. However, recent studies have shown that these herbs have multiple effects. For instance, *Ephedrae Herba* has antiallergic, antiasthmatic, anticoagulant, bronchodilator, smooth muscle relaxant, and vasoconstrictor activities [20–22]; *Pogostemonis (Agastachis) Herba* shows antiemetic, antiviral (influenza), antitumor, and smooth muscle relaxant activities [20, 23]; *Zingiberis Rhizoma Crudus* does analgesic, antibacterial, antiemetic, antimutagenic, antiulcer, hepatoprotective, and smooth muscle relaxant activities [20, 24–26]; and *Caryophylli Flos *does analgesic, anticoagulant, antiulcer, and smooth muscle relaxant activities [20, 27, 28]. Nevertheless, the fours herbs have shown to have anti-inflammatory activity in common. 

Although it is not fully understood how CP exerts its anti-inflammatory activity, it appears that CP targets NF-*κ*B. NF-*κ*B is a protein complex that promotes inflammation by expressing Proinflammatory cytokines and is found ubiquitously in most cells including lung parenchymal cells [29]. Aberrant NF-*κ*B activity has been known to be associated with many inflammatory diseases including inflammatory bowel disease [30], arthritis [31], sepsis [32], gastritis [33], asthma [34], COPD [34], and atherosclerosis [5]. In addition, the link between NF-*κ*B and inflammation in septic ALI has well been documented [35]. Thus, regulation of NF-*κ*B activity has been regarded as a reasonable therapeutic target for these inflammatory diseases. Two constituents of CP, *Ephedrae Herba* and *Zingiberis Rhizoma Crudus*, are known to suppress NF-*κ*B activity in RAW 264.7 macrophages stimulated with LPS [36, 37], and the other two constituents, *Pogostemonis (Agastachis) Herba* and *Caryophylli Flos*, suppress cytokine production in LPS-stimulated murine macrophages [38, 39]. Although precise mechanisms, by which the herbs suppress NF-*κ*B, remain unknown, it is presumable that CP, composed of the four herbs, suppresses NF-*κ*B. Indeed, our results show that CP suppressed NF-*κ*B activity and the production of Proinflammatory cytokines driven by NF-*κ*B in RAW 264.7 cells and in the lung.

Nrf2 has been found abundantly in tissues and organs that bear a high level of oxidative stress, such as lungs, liver, brain, GI tract, kidney, spleen, heart, and muscles [40, 41]. Recent studies have shown that Nrf2 is critical to fend the cytotoxic effects of oxidative stress [42] and plays an important role in regulating lung inflammation [40, 41]. These studies suggest that Nrf2 is an emerging therapeutic target against inflammatory diseases. Our results show that CP activated Nrf2, activating the expression of Nrf2 dependent genes in RAW 264.7 cells and in the lung. These results indicate that CP activates Nrf2 and its regulatory genes, contributing to anti-inflammatory function of CP. 

Given our results that CP activated Nrf2, it is conceivable that Nrf2 activated by CP suppresses NF-*κ*B, contributing to the anti-inflammatory effect of CP. Supportive to this notion, it has been reported that Nrf2 can directly suppress the functions of NF-*κ*B and AP-1, resulting in reduced expression of Proinflammatory cytokines elicited by LPS [43, 44]. However, sulforaphane, a potent activator of Nrf2, suppresses the expression of Proinflammatory cytokines by preventing oligomerization of TLR4 triggered by LPS [45], suggesting that Nrf2 may not directly suppress NF-*κ*B. In addition, neither overexpression of Nrf2 nor activation of Nrf2 by kaurenoic acid suppresses the expression of representative Proinflammatory genes including IL-1*β* and TNF-*α* in RAW 264.7 cells [46]. Although strongly activating Nrf2, the fruit hull of *Gleditsia sinensis* does not affect the function of NF-*κ*B in RAW 264.7 cells [15]. Although these results cannot exclude the possibility that CP suppresses NF-*κ*B via Nrf2, the impact of Nrf2 on NF-*κ*B activity remains to be elucidated. Nevertheless, our results suggest that CP has a potent anti-inflammatory activity by both suppressing NF-*κ*B and activating Nrf2. 

As described above, there are a plethora of reports suggesting that the four herbs composed of CP have anti-inflammatory functions. However, these studies mostly reported a preventive, rather than a therapeutic, effect of the herbs, because, in most cases, the herbs were treated prior to the onset of inflammatory response. In this study, unlike those studies, we attempted to address whether CP has a therapeutic effect on inflammatory lung disease. In addition, since CP is in use as an inhalant in clinic, we would like to retain a clinical relevancy by delivering CP in aerosol to the lungs of mice. To this end, we first injected LPS to the mice to induce ALI and thereby lung inflammation, and 2 h later delivered CP in aerosol directly to the lung using a micro-sprayer. According to our assessment, we routinely deliver CP to more than 80% of the lung (data not shown). Our results show that CP was highly effective in reducing neutrophilic infiltration to the lung incurred by ALI, suggesting that CP is a fast-acting therapeutics against acute lung inflammation, such as ALI. In addition, CP posttreatment to ALI mice suppressed the expression of Proinflammatory cytokines in the lung, accompanied by increased expression of Nrf2 dependent genes, which was consistent with our results with RAW 264.7 cells. Therefore, our results suggest that the therapeutic effect of CP on ALI is at least in part attributed to suppression of NF-*κ*B activity and activation of Nrf2 activity, which may be served as an underlying mechanism for the therapeutic effect of CP on ALI.

## 5. Conclusion

Here, we provide experimental evidence that CP had a therapeutic effect on ALI by using mice, which was mediated by differential regulation of the activities of NF-*κ*B and Nrf2. Our findings suggest that CP can be a new therapeutic formula against ALI. In addition, our study suggests the possibility that inhalation of ethnic herbal medicine is an administration route for the treatment of acutely developing inflammatory lung diseases, such as ALI.

## Figures and Tables

**Table 1:** Composition of Chung-pae.

Scientific name	Weight (g)
*Ephedrae Herba *	2
*Pogostemonis (Agastachis) Herba *	2
*Caryophylli Flos *	1
*Zingiberis Rhizoma Crudus *	1

Total	6

## References

[1] Irwin RS, Rippe JM (2003). *Irwin and Rippe's Intensive Care Medicine*.

[2] Zhu YG, Qu JM, Zhang J, Jiang HN, Xu JF (2011). Novel interventional approaches for ALI/ARDS: cell-based gene therapy. *Mediators of Inflammation*.

[3] Devaney J, Contreras M, Laffey JG (2011). Clinical review: gene-based therapies for ALI/ARDS: where are we now?. *Critical Care*.

[4] Tolle LB, Standiford TJ (2013). Danger-associated molecular patterns (DAMPs) in acute lung injury. *The Journal of Pathology*.

[5] Monaco C, Andreakos E, Kiriakidis S (2004). Canonical pathway of nuclear factor *κ*B activation selectively regulates proinflammatory and prothrombotic responses in human atherosclerosis. *Proceedings of the National Academy of Sciences of the United States of America*.

[6] Wheeler AP, Bernard GR (1999). Treating patients with severe sepsis. *The New England Journal of Medicine*.

[7] Rangasamy T, Cho CY, Thimmulappa RK (2004). Genetic ablation of Nrf2 enhances susceptibility to cigarette smoke-induced emphysema in mice. *Journal of Clinical Investigation*.

[8] Rangasamy T, Guo J, Mitzner WA (2005). Disruption of Nrf2 enhances susceptibility to severe airway inflammation and asthma in mice. *Journal of Experimental Medicine*.

[9] Sussan TE, Rangasamy T, Blake DJ (2009). Targeting Nrf2 with the triterpenoid CDDO-imidazolide attenuates cigarette smoke-induced emphysema and cardiac dysfunction in mice. *Proceedings of the National Academy of Sciences of the United States of America*.

[10] Boutten A, Goven D, Artaud-Macari E, Boczkowski J, Bonay M (2011). NRF2 targeting: a promising therapeutic strategy in chronic obstructive pulmonary disease. *Trends in Molecular Medicine*.

[11] Chen JK, Chen TT (2009). *Chinese Herbal Formulas and Applications: Pharmacological Effects & Clinical Research*.

[12] Lyu JH, Kim KH, Kim HW (2012). Dangkwisoo-san, an herbal medicinal formula, ameliorates acute lung inflammation via activation of Nrf2 and suppression of NF-*κ*B. *Journal of Ethnopharmacology*.

[13] Thimmulappa RK, Lee H, Rangasamy T (2006). Nrf2 is a critical regulator of the innate immune response and survival during experimental sepsis. *Journal of Clinical Investigation*.

[14] Chan K, Kan YW (1999). Nrf2 is essential for protection against acute pulmonary injury in mice. *Proceedings of the National Academy of Sciences of the United States of America*.

[15] Choi JY, Kwun MJ, Kim KH (2012). Protective effect of the fruit hull of Gleditsia sinensis on LPS-induced acute lung injury is associated with Nrf2 activation. *Evidence-Based Complementary and Alternative Medicine*.

[16] Johnson JA, Johnson DA, Kraft AD (2008). The Nrf2-ARE pathway: an indicator and modulator of oxidative stress in neurodegeneration. *Annals of the New York Academy of Sciences*.

[17] Venugopal R, Jaiswal AK (1996). Nrf1 and Nrf2 positively and c-Fos and Fra1 negatively regulate the human antioxidant response element-mediated expression of NAD(P)H:quinone oxidoreductase1 gene. *Proceedings of the National Academy of Sciences of the United States of America*.

[18] Solis WA, Dalton TP, Dieter MZ (2002). Glutamate-cysteine ligase modifier subunit: mouse Gclm gene structure and regulation by agents that cause oxidative stress. *Biochemical Pharmacology*.

[19] Bensky D, Clavey S, Stöger E (2004). *Chinese Herbal Medicine: Materia Medica*.

[20] Chen JK, Chen TT, Crampton L (2004). *Chinese Medical Herbology and Pharmacology*.

[21] Liu YG, Luo JB (2007). Effects of among compositions of Herba Ephedrae decoction on genic xpression of 5-lipoxygenase activating protein, IL-4 and leukotriene C 4 in asthmatic mice. *Zhongguo Zhongyao Zazhi*.

[22] Zhai HQ, Zhang SF, Gao MC (2011). Effects of herba Ephedra sinicae and fructus schisandrae Chinensis on pathology of rats with bleomycin A5-induced idiopathic pulmonary fibrosis. *Zhong Xi Yi Jie He Xue Bao*.

[23] Li YC, Peng SZ, Chen HM (2012). Oral administration of patchouli alcohol isolated from Pogostemonis Herba augments protection against influenza viral infection in mice. *International Immunopharmacology*.

[24] Aimbire F, Penna SC, Rodrigues M, Rodrigues KC, Lopes-Martins RAB, Sertié JAA (2007). Effect of hydroalcoholic extract of Zingiber officinalis rhizomes on LPS-induced rat airway hyperreactivity and lung inflammation. *Prostaglandins Leukotrienes and Essential Fatty Acids*.

[25] Kuo PL, Hsu YL, Huang MS, Tsai MJ, Ko YC (2011). Ginger suppresses phthalate ester-induced airway remodeling. *Journal of Agricultural and Food Chemistry*.

[26] Ghayur MN, Gilani AH, Janssen LJ (2008). Ginger attenuates acetylcholine-induced contraction and Ca^2+^ signalling in murine airway smooth muscle cells. *Canadian Journal of Physiology and Pharmacology*.

[27] Magalhães CB, Riva DR, Depaula LJ (2010). In vivo anti-inflammatory action of eugenol on lipopolysaccharide-induced lung injury. *Journal of Applied Physiology*.

[28] Saini A, Sharma S, Chhibber S (2009). Induction of resistance to respiratory tract infection with *Klebsiella pneumoniae* in mice fed on a diet supplemented with tulsi (*Ocimum sanctum*) and clove (*Syzgium aromaticum*) oils. *Journal of Microbiology, Immunology and Infection*.

[29] Gilmore TD (2006). Introduction to NF-*κ*B: players, pathways, perspectives. *Oncogene*.

[30] Atreya I, Atreya R, Neurath MF (2008). NF-*κ*B in inflammatory bowel disease. *Journal of Internal Medicine*.

[31] Makarov SS (2001). NF-*Κ*B in rheumatoid arthritis: a pivotal regulator of inflammation, hyperplasia, and tissue destruction. *Arthritis Research*.

[32] Abraham E (2003). Nuclear factor-*κ*B and its role in sepsis-associated organ failure. *Journal of Infectious Diseases*.

[33] Moorchung N, Srivastava AN, Sharma AK, Achyut BR, Mittal B (2010). Nuclear factor *κ*-B and histopathology of chronic gastritis. *Indian Journal of Pathology & Microbiology*.

[34] Edwards MR, Bartlett NW, Clarke D, Birrell M, Belvisi M, Johnston SL (2009). Targeting the NF-*κ*B pathway in asthma and chronic obstructive pulmonary disease. *Pharmacology and Therapeutics*.

[35] Schwartz MD, Moore EE, Moore FA (1996). Nuclear factor-*κ*B is activated in alveolar macrophages from patients with acute respiratory distress syndrome. *Critical Care Medicine*.

[36] Kim IS, Park YJ, Yoon SJ, Lee HB (2010). Ephedrannin A and B from roots of *Ephedra sinica* inhibit lipopolysaccharide-induced inflammatory mediators by suppressing nuclear factor-*κ*B activation in RAW 264.7 macrophages. *International Immunopharmacology*.

[37] Lee HY, Park SH, Lee M (2012). 1-Dehydro-[10]-gingerdione from ginger inhibits IKK*β* activity for NF-*κ*B activation and suppresses NF-*κ*B-regulated expression of inflammatory genes. *The British Journal of Pharmacology*.

[38] Xian YF, Li YC, Ip SP, Lin ZX, Lai XP, Su ZR (2011). Anti-inflammatory effect of patchouli alcohol isolated from Pogostemonis Herba in LPS-stimulated RAW264.7 macrophages. *Experimental and Therapeutic Medicine*.

[39] Bachiega TF, de Sousa JPB, Bastos JK, Sforcin JM (2012). Clove and eugenol in noncytotoxic concentrations exert immunomodulatory/anti-inflammatory action on cytokine production by murine macrophages. *Journal of Pharmacy and Pharmacology*.

[40] Lee JM, Li J, Johnson DA (2005). Nrf2, a multi-organ protector?. *FASEB Journal*.

[41] Cho HY, Reddy SP, Kleeberger SR (2006). Nrf2 defends the lung from oxidative stress. *Antioxidants and Redox Signaling*.

[42] Gold R, Kappos L, Arnold DL (2012). Placebo-controlled phase 3 study of oral BG-12 for relapsing multiple sclerosis. *The New England Journal of Medicine*.

[43] Li W, Khor TO, Xu C (2008). Activation of Nrf2-antioxidant signaling attenuates NF*κ*B-inflammatory response and elicits apoptosis. *Biochemical Pharmacology*.

[44] Liu GH, Qu J, Shen X (2008). NF-*κ*B/p65 antagonizes Nrf2-ARE pathway by depriving CBP from Nrf2 and facilitating recruitment of HDAC3 to MafK. *Biochimica et Biophysica Acta*.

[45] Youn HS, Kim YS, Park ZY (2010). Sulforaphane suppresses oligomerization of TLR4 in a thiol-dependent manner. *Journal of Immunology*.

[46] Lyu JH, Lee GS, Kim KH (2011). Ent-kaur-16-en-19-oic acid, isolated from the roots of Aralia continentalis, induces activation of Nrf2. *Journal of Ethnopharmacology*.

